# Immunomodulatory effects of oral microbiota in the pathogenesis of rheumatoid arthritis

**DOI:** 10.3389/fimmu.2026.1707949

**Published:** 2026-02-06

**Authors:** Yiming Yang, Guanyuan Wang, Yongzhou Song, Jun Ma, Aijing Liu

**Affiliations:** 1 Department of Rheumatology and Immunology, The Second Hospital of Hebei Medical University, Shijiazhuang, Hebei, China; 2 Hebei Medical University-National University of Ireland Galway Stem Cell Research Center, Hebei Medical University, Shijiazhuang, Hebei, China; 3 Hebei International Joint Research Center on Rheumatic Diseases, Shijiazhuang, Hebei, China; 4 Department of Orthopedics, The Second Hospital of Hebei Medical University, Shijiazhuang, Hebei, China; 5 Hebei Research Center for Stem Cell Medical Translational Engineering, Shijiazhuang, Hebei, China; 6 Department of Anatomy, Hebei Medical University, Shijiazhuang, Hebei, China

**Keywords:** rheumatoid arthritis, periodontal disease, oral microbiota, microbial dysbiosis, Leukotoxin A, peptidylarginine deiminase

## Abstract

Rheumatoid arthritis (RA) is a chronic autoimmune disease characterized by persistent synovial inflammation, progressive cartilage and bone destruction, and resulting functional disability. Its pathogenesis is multifactorial, involving both genetic predisposition and environmental influences. In recent years, the interaction between the oral microbiota and RA has emerged as a prominent research focus. Dysbiosis of the oral microbiome, defined as an imbalance in microbial composition relative to a healthy state, accompanies disease onset and may further act as a trigger of systemic autoimmune responses. Specific virulence factors, including the peptidylarginine deiminase from *Porphyromonas gingivalis* and leukotoxin A from *Aggregatibacter actinomycetemcomitans*, promote excessive protein citrullination and anti-citrullinated protein antibody generation, thereby contributing to the loss of immune tolerance, particularly in genetically susceptible individuals. Moreover, the bidirectional relationship between RA and periodontitis highlights shared inflammatory pathways that contribute to both periodontal and joint tissue destruction. Potential mechanisms include bacteremia induced by routine oral activities, systemic dissemination of bacterial products, and colonization of oral microbiota in the gastrointestinal tract. Current evidence suggests that periodontal therapy may reduce systemic inflammatory markers and occasionally improve RA activity, although results remain inconsistent. In this review, we explored the potential mechanisms underlying the imbalance of the oral microbiota and its contribution to the onset and progression of RA, focusing on microbially induced citrullination, host genetic susceptibility, and common inflammatory pathways, while also discussing the impact of comprehensive periodontal management and lifestyle interventions on RA outcomes. Overall, these insights underscore the role of the oral microbiome in RA pathogenesis and suggest that addressing microbial dysbiosis through integrated therapeutic strategies may complement conventional care.

## 1 Introduction

Rheumatoid arthritis (RA) is an autoimmune disease characterized by chronic joint inflammation, with typical manifestations including joint swelling and pain, morning stiffness, extra-articular involvement, and autoantibody production, which may eventually result in joint deformities, disability, and internal organ failure (1). Although the mechanism of RA remains indecisive, RA is considered a complex multifactorial disease, including genetic susceptibility, environmental factors, infections, hormone dysregulation, smoking, and diet, among which genetic and environmental factors are critically involved in the pathogenesis of RA (2). There has been a growing focus on the role of oral and intestinal microbiota in the pathogenesis of RA in recent years.

The oral cavity serves as the entry point to the digestive system, and its microbiota represents the second largest microbial community in the human body, following the gut microbiota (3). This complex microbial community colonizes teeth, restorative surfaces, and mucosal surfaces within surface-attached communities known as dental plaque (4). These microorganisms form complex communities in the form of biofilms and perform the physiological functions of microbes. When in homeostasis with the host, the oral microbial community acts as a physiological barrier against invasion by exogenous pathogens, while the imbalanced ecological relationship with the host, various chronic infectious diseases such as dental caries, periapical periodontitis, periodontal disease (PD), pericoronitis of wisdom teeth and osteomyelitis of jawbone, etc, severely threaten oral health, even triggering autoimmune diseases (5).

PD is an inflammatory condition caused by the host’s immune response to microbial biofilm formation (6). The pathogenesis of this disease is complex, involving pathogens, symbionts, and host oral immune dysfunction. PD has been identified as a risk factor for a range of systemic conditions, including diabetes, cardiovascular and respiratory diseases, adverse pregnancy outcomes, and rheumatic disorders such as RA (7). Studies have shown that there is a higher prevalence of PD in patients with RA (8). The connection between RA and PD might stem from shared environmental factors, like smoking, and genetic predispositions. There are notable similarities between RA and PD at multiple levels, making the comorbidity of these diseases potentially more harmful to the patient’s overall health. Additionally, RA may increase the susceptibility to developing PD. Through various pathways, including the activity of periodontal bacteria, PD may also be a risk factor for RA (9). In particular, specific periodontal pathogens express virulence factors capable of directly or indirectly promoting key cellular mechanisms implicated in RA pathogenesis, including protein citrullination, activation of the NOD-like receptor family pyrin domain containing 3 (NLRP3) inflammasome and immune evasion strategies that enable persistence despite host defense responses (10). Understanding this interplay offers promising opportunities for early diagnosis, identification of high risk individuals, and integrated treatment strategies. Accordingly, this review summarizes current evidence from peer-reviewed clinical and experimental studies investigating the link between PD, oral microbiota, and RA, while excluding conference abstracts and non-original reports.

## 2 Immunological mechanisms linking oral microbiota to RA

### 2.1 Immunopathogenesis of RA

RA is a multifactorial autoimmune disease characterized by persistent synovial inflammation, autoantibody production, and progressive joint destruction (11). Its pathogenesis arises from complex interactions among innate and adaptive immunity, genetic susceptibility, and environmental triggers, with the oral microbiota emerging as a potential initiator and amplifier of autoimmunity (12). Innate immune cells, such as macrophages, dendritic cells (DCs), neutrophils, and natural killer (NK) cells, are activated early in RA and initiate synovial inflammation (13). Activated antigen-presenting cells (APCs) release pro-inflammatory cytokines, including tumor necrosis factor-α (TNF-α), interleukin (IL)-1β, IL-6, IL-18, IL-23, and reactive oxygen species (ROS), thereby amplifying local immune responses and recruiting effector cells (14). Subsequently, neutrophils release enzymes that can break down the matrix and form neutrophil extracellular traps (NETs), which leads to joint damage and exposes self-antigens, especially citrullinated proteins (15). Consistent with this mechanism, Leukotoxin A (LtxA) from *Aggregatibacter actinomycetemcomitans (A. actinomycetemcomitans)* induces excessive citrullination and NET-like release from human neutrophils through dysregulated peptidylarginine deiminase (PPAD) activation, potentially contributing to autoantigen generation in RA (16).

**Figure 1:**
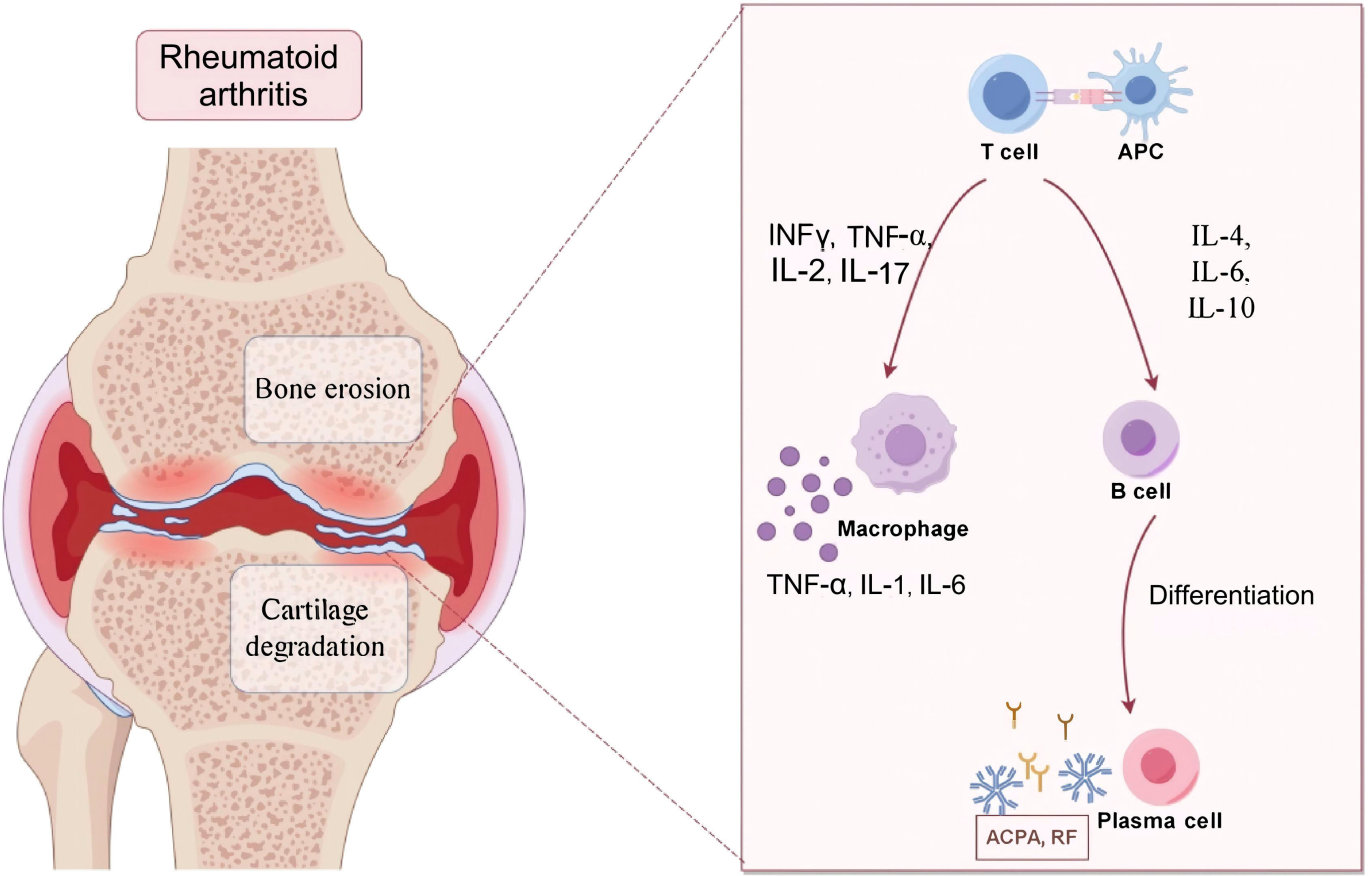
Pathogenesis of RA. Created in https://www.figdraw.com.

Toll-like receptors (TLRs) such as TLR-2, TLR-4, and TLR-9 recognize microbial components like lipopolysaccharides (LPS) and peptidoglycan (17). In RA synovium, abnormal TLR activation, particularly in response to oral pathogens, sustains chronic inflammation through cytokine overproduction and oxidative stress (18). This highlights a mechanistic link between oral microbial dysbiosis and aberrant innate immune activation in RA. Adaptive immunity is central in sustaining RA. CD4^+^ T cells, particularly Th17, secrete IL-17, TNF-α, IL-21, and IL-22, driving synovial hyperplasia, cartilage destruction, and osteoclastogenesis (19). A Th17/Treg imbalance perpetuates autoimmunity. B cells act beyond antibody secretion by presenting antigens, producing cytokines, forming ectopic germinal centers, and generating rheumatoid factor (RF) and anti-citrullinated protein antibodies (ACPAs) (20). These autoantibodies form immune complexes that activate complement cascades, aggravating joint injury. Thus, both T and B cells drive RA initiation and persistence (**Figure 1**).

Genetically, the strongest risk locus for RA is the human leukocyte antigen (HLA) class II region, particularly HLA-DRB1 alleles encoding the shared epitope (SE), which enhances the presentation of citrullinated peptides to autoreactive CD4^+^ T cells, predisposing individuals to ACPA positive (ACPA^+^) RA (21). This directly connects host genetics to microbial and environmental factors. For instance, *Porphyromonas gingivalis (P. gingivalis)* expresses a unique bacterial PPAD that citrullinates both bacterial and host proteins (22). These neoantigens are preferentially presented by SE-positive HLA-DR molecules, promoting loss of tolerance and ACPA generation (23). Thus, the HLA-DRB1 SE citrullination axis provides a molecular bridge linking dysbiotic oral microbiota to systemic autoimmunity in RA. These observations suggest that greater emphasis should be placed on virulence factors unique to periodontal pathogens, including PPAD and LtxA, as potential microbial determinants linking oral dysbiosis to RA-specific autoimmune responses.

### 2.2 Oral microbiota and ACPA generation

**Figure 2:**
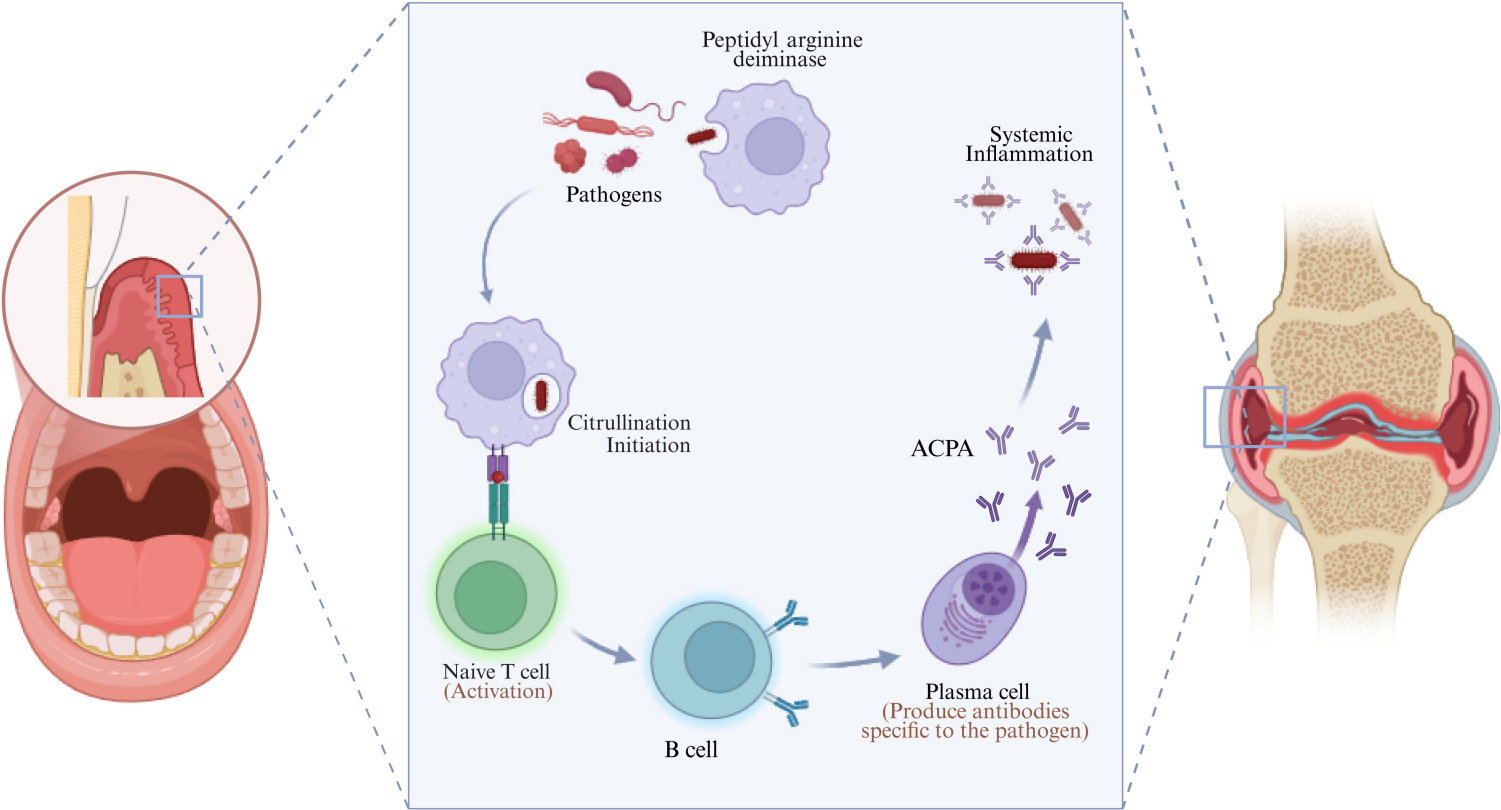
Biological intersections between oral microbiota and RA. In the periodontitis environment, bacteria promote citrullination of host proteins through direct or indirect mechanisms, generating antigens misrecognized by the immune system, and thereby inducing the production of RA related autoantibodies (ACPA), triggering the immune cascade reaction of RA. Created in https://BioRender.com.

ACPAs are a hallmark of RA and provide a critical link between oral bacteria and systemic autoimmunity. Oral pathogens can trigger protein citrullination, converting arginine residues into citrulline and thereby stimulating ACPA production. ACPAs can be detected years before the onset of clinical RA, underscoring their value as predictive biomarkers (24). These antibodies contribute to joint pathology while simultaneously amplifying systemic inflammation, impacting RA progression (**Figure 2**). In ACPA^+^ RA patients, immune dysregulation is more pronounced, leading to severe inflammation and tissue destruction in both joints and periodontal tissues. Elevated levels of TNF-α and IL-6 may drive parallel inflammatory cascades in synovium and gingiva (25). In contrast, ACPA negative (ACPA^-^) RA is often associated with milder periodontal manifestations, likely due to reduced citrullination and more localized immune activation. Mechanistic studies indicate that *P. gingivalis* promotes ACPA generation through epitope mimicry and uptake by APCs (26). A case report by Mukherjee et al. described a patient with concomitant RA and periodontitis in whom successful eradication of a highly leukotoxic JP2 genotype *A. actinomycetemcomitans* infection was associated with sustained remission of RA symptoms, further supporting a potential causal link between periodontal pathogens and systemic autoimmune activity (27). Periodontal therapy in ACPA^+^ patients has been shown to reduce systemic inflammation and potentially slow disease progression (28).

### 2.3 Microbial PADs and citrullination: the molecular connection

Citrullination, a PPAD catalyzed modification, is central to RA pathogenesis. PPADs in immune cells such as lymphocytes, neutrophils, and macrophages convert arginine to citrulline, generating neoantigens that elicit anti-cyclic citrullinated peptide or ACPA production (29). In RA, its excessive citrullination is strongly associated with autoantibody production and marks early disease onset. *P. gingivalis* is the only known microorganism capable of producing bacterial PPAD, an enzyme that mimics host PPAD activity and facilitates the generation of citrullinated bacterial and host proteins (30). Beyond PPAD-dependent citrullination, A. actinomycetemcomitans can also promote protein citrullination through a distinct mechanism, as its LtxA induces aberrant activation of host PPAD in neutrophils, resulting in the accumulation of hypercitrullinated antigens (31). Furthermore, animal studies show that inhibiting PPAD with small molecules or antibodies reduces *P. gingivalis* induced citrullination and mitigates RA severity (32). Clinical studies have shown that oral infections are associated with the systemic autoimmune response of RA, and periodontitis caused by *P. gingivalis* is associated with elevated serum anti-cyclic citrullinated peptide antibodies. This indicates that the antigens produced by PD induced PPAD may trigger synovial inflammation and autoimmune responses (33). PPAD derived peptides can engage fragment crystallizable receptors and complement receptors in synovial tissue, leading to local inflammation and joint destruction. Periodontitis prevalence correlates with ACPA^+^ in RA, while citrullinated protein accumulation links to aggressive disease and early bone erosion (34). The citrullination process triggered by oral microbiota may potentially serve as a certain molecular link between periodontitis and RA. The interplay of microbial PADs, host immunity, and genetic susceptibility presents a potential therapeutic target, with PPAD inhibition or oral microbiome modulation offering promising strategies to curb RA development.

### 2.4 Biological intersection between periodontitis and RA

Periodontitis is a chronic inflammatory disease that affects the gingiva and tooth-supporting structures. Epidemiological studies demonstrate that RA patients are up to two times more likely to develop periodontitis than healthy controls (35). Moreover, a bidirectional relationship appears to exist: systemic inflammation and immune dysregulation in RA predispose to periodontitis, while chronic periodontal infection and inflammation exacerbate RA onset and activity by triggering systemic immune responses. RA alters the oral environment through impaired immune regulation, which favors pathogenic bacterial overgrowth. Additionally, xerostomia, often observed in RA patients due to Sjögren’s syndrome or long term use of immunosuppressive agents (e.g., methotrexate, corticosteroids), reduces salivary flow and antimicrobial protection, thereby increasing susceptibility to oral dysbiosis and periodontitis (36). Conversely, systemic inflammation initiated by PD can aggravate RA symptoms (37). These research findings collectively indicate that there is a biological connection, namely that the inflammation triggered by the oral microbiota can promote the onset process of RA (**Table 1**).

**Table 1:** A contribution of oral microbiota to RA in clinical and experimental studies.

Author, year	Region	Study group (N)	Sample	Microbiome assessment method	Finding
Kozhakhmetov, S.et al., 2023 (87)	Kazakhstan	RA and HC (75/114)	Saliva	16S rRNA gene sequencing	RA patients showed increased oral microbial diversity and higher abundance of *Prevotellaceae* and *Leptotrichiaceae*, with specific taxa correlating with disease activity and autoantibody levels.
Eriksson, K.et al., 2022 (88)	Sweden	RA combined with periodontitis and HC (53/48)	Saliva	16S rRNA gene sequencing(V3-V4)	RA patients exhibiting higher abundances of *Granulicatella*, *Veillonella*, *Megasphaera*, and *F. nucleatum*, alongside elevated levels of inflammatory mediators like pentraxin-3, and IL-19.
Lehenaff, R.et al., 2021 (97)	USA	RA and HC (8/10)	Subgingival plaque	16S rRNA gene sequencing (V1-V3)	The differences in the subgingival microbiota in the deep and shallow pockets of RA patients were smaller and their structures were more similar. Moreover, *Streptococcus parasanguinis* and *Actinomyces meyeri* were enriched in RA.
Muñoz-Atienza, E. et al., 2020 (61)	UK	ERA and HC (10/8)	Serum	16S rRNA gene sequencing	*P. gingivalis* contributes to RA pathogenesis through gut barrier disruption and joint inflammation, but its autocitrullinated proteins are not major ACPA targets.
Corrêa, J D. et al., 2019 (91)	Brazil	RA combined with periodontitis and HC (42/47)	Supragingival plaque	16S rRNA gene sequencing (V4-V5)	RA subjects exhibited significantly greater periodontal destruction. Additionally, the majority of RA patients with periodontitis (85%) tested positive for ACPA.
Lopez-Oliva, I. et al., 2018 (89)	UK	RA and HC (22/19)	Subgingival plaque	16S rRNA gene sequencing	The enrichment of *P. gingivalis* and *Cryptobacterium curtum* in RA patients, as well as the reduction of commensal bacteria, this imbalance has already emerged even in cases of clinically healthy periodontal conditions.
Mukherjee, A. et al., 2018 (27)	USA	1 RA patient with refractory seropositive RA and A. actinomycetemcomitans endocarditis (case)	Serum, blood cultures, neutrophils	PCR/Sanger sequencing; ELISA	Genetic susceptibility and elevated anti-LtxA and cytokines indicated a mechanism linking *A. actinomycetemcomitans* infection to RA autoimmunity.
Konig MF et al., 2016 (16)	USA	PD and HC (118/108)	Gingival crevicular fluid, neutrophils	Proteomic, PCR, ELISA	Periodontitis gingival crevicular fluid shows patterns of hypercitrullination similar to RA joint. *A. actinomycetemcomitans* induces neutrophil hypercitrullination via LtxA; exposure to leukotoxic strains is associated with ACPAs and RF in RA patients.

N, Number of patients; HC, Healthy control.

### 2.5 Biological intersections between oral microbiota and RA

Certain environmental and genetic factors, including smoking, genetic predisposition (e.g., HLA-DRB1 SE), alterations in the oral microbiome, and microbial infections, can promote post-translational modifications of proteins, such as citrullination. In genetically susceptible individuals, these modifications, together with local inflammatory responses mediated by macrophages, DCs, and T lymphocytes, may trigger an immune response against citrullinated proteins (38). Activated immune cells subsequently release a spectrum of proinflammatory mediators, including ILs, prostaglandins, TNF, and matrix metalloproteinases (MMPs), which further amplify the inflammatory cascade and tissue destruction. Among these, IL-17, a key cytokine of the Th17 axis, induces MMP and ROS production, while also stimulating osteoclast differentiation indirectly through upregulation of receptor activator of nuclear factor kappa-B ligand (RANKL) expression on osteoblasts (39).

Reactive lymphocytes, particularly B cells and helper T cell subsets (Th1 and Th17), are key factors that drive osteoclasts to undergo bone resorption through a mechanism dependent on RANKL. This process is observed in two interrelated pathological contexts: In RA, autoreactive T cells (notably Th1 and Th17) secrete proinflammatory cytokines that enhance RANKL expression, leading to the differentiation and activation of osteoclast precursors and ultimately causing erosive bone destruction within the joints (40); In PD, the host immune response to periodontal pathogens similarly involves B and Th17 cells, which promote elevated RANKL expression and subsequent osteoclastogenesis, resulting in alveolar bone loss surrounding teeth (41). These parallel mechanisms highlight a shared immunopathological axis between RA and PD, mediated through aberrant lymphocyte activation and dysregulated bone remodeling. Beyond RANKL-driven mechanisms, activation of the NLRP3 inflammasome provides an additional link between immune dysregulation and bone loss in both RA and PD. NLRP3-dependent maturation of IL-1β in macrophages and neutrophils promotes osteoclast differentiation by enhancing RANKL signaling and amplifying Th17-mediated inflammation (42). Increased NLRP3 and IL-1β activity has been associated with erosive joint damage in RA, while similar pathways contribute to inflammation induced alveolar bone loss in periodontitis (43). Furthermore, an increasing amount of evidence indicates the existence of a bidirectional relationship. RA may exacerbate periodontal inflammation, while the imbalance of oral microbiota in turn can worsen the progression of RA.

## 3 Pathogenic oral bacteria implicated in RA

Recent studies have demonstrated a strong link between the composition of oral microbiota and the development of RA. Several periodontal pathogens can promote systemic inflammation and immune dysregulation, potentially triggering autoimmune responses through mechanisms such as molecular mimicry, immune evasion, and the induction of citrullinated proteins. This section summarizes the key bacterial species implicated in this association and discusses their potential roles in RA pathogenesis (**Table 2**).

**Table 2:** The mechanism relationship between Key oral pathogenic bacteria and RA.

Periodontal pathogen	Mechanism	Pathway involved	Reference
*Porphyromonas gingivalis*	Secretes virulence factors (LPS, gingipains, collagenase) disrupting periodontal tissue	PAD–citrullination–ACPA TLR2/4 pathway IL-8 degradation pathway	Svärd, Anna., et al (47)
*Aggregatibacter actinomycetemcomitans*	Produces LtxA, LPS, adhesins, exotoxins disrupting host defenses	LtxA–NETs–citrullination axis CD11/CD18 signaling pathway	Johansson A., et al (49)
*Prevotella intermedia*	Induce IL-1β, IL-8, MIP, proteases, and MMPs to promoting inflammation and tissue breakdown	PAD–citrullination pathway Neutrophil activation and immune evasion (nucA/nucD-mediated)	Zhang, S., et al (51)
*Fusobacterium nucleatum*	Activates NK cells to trigger periodontal inflammation	TLR2 and TLR4 signaling pathway IL-6,IL-8, TNF-α cytokine axis	Meng, Q., et al (54)
*Tannerella forsythia*	Produces BspA adhesin, facilitates co-aggregation with *F. nucleatum*	TLR2-mediated immune activation IL-6/TNF-α signaling axis	Martínez-Rivera, J., et al (58)

### 3.1 Porphyromonas gingivalis

The Periodontal *P. gingivalis* is a pathogenic bacterium that mainly causes human periodontitis. There are multiple ways for *P. gingivalis* to increase bacterial colonization and disrupt the host’s periodontal structure, including the production of LPS, gingipains, and collagenases (44). Furthermore, *P. gingivalis* virulence factors can enhance adhesion and biofilm accumulation with other bacteria, as well as regulate or interfere with inflammatory responses to evade host immune reactions. Specifically, *P. gingivalis* invades host cells using its fimbriae, and mutations in the fimbriae-A subunit protein encoded by its gene affect the ability to form biofilms (45). Moreover, animal model experiments have found that *P. gingivalis* strains lacking major fimbrial proteins are unable to bind to oral surfaces or cause alveolar bone loss (46). In addition, *P. gingivalis* expressing PPAD enzymes may induce ACPA production and promote RA development, which is associated with anti-*P. gingivalis* titers in RA patients, they usually exhibit higher titers of anti-*P. gingivalis* antibodies (47). The presence of this high titer antibody indicates that *P. gingivalis* may significantly contribute to RA’s pathogenesis through its PPAD enzyme activity, facilitating autoimmune reactions and advancing the disease progression. These results suggest that *P. gingivalis* may affect the body’s immune system by altering TLR responses, damaging IL-8, or altering complement cascade reactions.

### 3.2 Aggregatibacter actinomycetemcomitans

*A. actinomycetemcomitans* is an anaerobic, gram negative coccus that colonizes the oral cavity and has been implicated in both aggressive and chronic periodontitis. This microorganism produces a range of virulence factors, including LPS, adhesins, biofilms, and exotoxins. Among them, LtxA is considered a key effector molecule that significantly impairs host immune function (48). LtxA promotes pathology by targeting leukocytes via β2 integrins, inducing ionic fluxes that drive NLRP3 inflammasome activation and subsequent caspase-1 dependent release of pro-inflammatory cytokines such as IL-1β and IL-18. Additionally, LtxA activates osteoclasts through the CD11/CD18 pathway and promotes NET formation, thereby contributing to tissue damage and systemic inflammation (49). The enhanced production of reactive oxygen species appears to function as a downstream amplifying signal rather than a pathogen-specific mechanism unique to periodontal bacteria. LtxA facilitates calcium influx in T cells, leading to calpain activation and recruitment of β2 integrins into lipid rafts, which contribute to immune cell activation. In neutrophils, LtxA induced calcium signaling also promotes PAD activation and histone citrullination, facilitating NET formation enriched in citrullinated autoantigens. *A. actinomycetemcomitans* periodontal infections may disrupt immune tolerance in RA by generating antigens targeted by RA autoantibodies. Its LtxA alters neutrophil morphology and boosts inflammatory mediator release from macrophages (50). The *A. actinomycetemcomitans* strains with leukocyte toxicity characteristics are more prevalent in patients with periodontitis and RA, and are associated with elevated levels of ACPAs, suggesting that they may be involved in the initiation or exacerbation of the autoimmune response in RA.

### 3.3 Prevotella intermedia

*Prevotella* spp., including *Prevotella Media* (*P. media*) and *Prevotella intermedia* (*P. intermedia*), are anaerobic gram-negative bacteria commonly enriched in the periodontitis associated microbiome. These species contribute to the onset and progression of periodontitis by inducing the release of pro-inflammatory mediators such as IL-1β, IL-8, macrophage inflammatory proteins, proteases, and MMPs (51). Emerging evidence suggests that *Prevotella* spp. may contribute to the initiation and amplification of RA pathogenesis. *P. media* has been shown to translocate into the systemic circulation, potentially reaching distant tissues such as the synovium. It may worsen RA by boosting systemic inflammation, activating neutrophils, and promoting PPAD activity, which drives protein citrullination, a key step in ACPA production and immune tolerance loss (52). These immune evasion strategies may prolong inflammation and support chronic synovitis, promoting synovial hyperplasia and osteoclast differentiation, two hallmark features of RA. In contrast, P. intermedia mainly affects the local periodontal environment by increasing MMP-1 and MMP-8 in ligament cells and promoting prostaglandin production through the arachidonic acid pathway (53). These actions aggravate periodontal damage and may systemically promote RA by elevating inflammatory mediators and enzymes that drive joint destruction and bone erosion.

### 3.4 Fusobacterium nucleatum

*Fusobacterium nucleatum* (*F. nucleatum*) is an anaerobic gram-negative bacterium predominantly colonizing periodontal pockets and has been implicated in the pathogenesis of both periodontitis and RA. Under physiological conditions, host immune homeostasis is maintained by a balance of pro- and anti-inflammatory cytokines. However, ecological dysbiosis or the translocation of *F. nucleatum* beyond the oral cavity can trigger systemic immune activation, disrupting this equilibrium (54). In early RA, *F. nucleatum* may trigger disease by activating NK cells and promoting pro-inflammatory cytokines, disrupting peripheral tolerance. It notably stimulates TLR4 signaling, a key innate immune pathway upregulated in RA leukocytes (55). Activation of TLR4 and TLR2 pathways promotes DC maturation and inflammatory cytokine production, contributing to the priming of autoreactive T cells and potentially triggering autoimmunity. In disease progression, *F. nucleatum* strongly stimulates the secretion of IL-6, IL-8, and TNF-α, which are key mediators in RA pathophysiology. These cytokines drive macrophage and synoviocyte activation, enhance synovial hyperplasia, and facilitate osteoclast differentiation and bone resorption (56). In particular, TNF-α promotes the expression of RANKL, which is essential for osteoclastogenesis, thus linking *F. nucleatum* induced inflammation directly to bone and cartilage destruction, a hallmark of RA.

### 3.5 Tannerella forsythia

*Tannerella forsythia* (*T. forsythia*) is an obligate anaerobic bacterium associated with periodontitis. Although lacking flagella, *T. forsythia* compensates through the production of Bacteroides surface protein A (BspA), an adhesin that interacts with *P. gingivalis* like domains and promotes co-aggregation with *F. nucleatum*. BspA activates monocytes and gingival epithelial cells via a TLR2-dependent pathway, inducing the release of IL-8 and other pro-inflammatory mediators (57). *T. forsythia* lipoproteins stimulate gingival fibroblasts and monocytes to release IL-6 and TNF-α, key cytokines in RA pathogenesis (58). TNF-α worsens inflammation by enhancing lymphocyte infiltration and upregulating IL-1β, IL-6 and MMPs, leading to cartilage and bone damage. IL-6 further promotes autoantibody production, endothelial activation, and osteoclastogenesis, accelerating joint erosion (59). However, it remains unclear if *T. forsythia* directly triggers RA onset, as it has not been proven to break immune tolerance or induce antigen specific autoimmunity like *P. gingivalis*. Therefore, the current evidence suggests that honeysuckle may act as a potential factor contributing to the progression of RA by enhancing the inflammatory response mediated by cytokines, rather than being the direct cause of the disease onset.

## 4 Causes of oral microbiota imbalance

### 4.1 Oral microbiota and autoimmunity

Studies using animal models have demonstrated that oral inoculation with *P. gingivalis* increases Th17 cell populations, induces bone loss, and exacerbates autoimmune arthritis, highlighting the role of oral microbiota in modulating host immunity and contributing to autoimmune disease development (60). Furthermore, the presence of SE enhances the percentage of Th17 cells upon inoculation with *P. gingivalis*, significantly increases bone loss, and makes it possible for ACPA production in the serum of arthritic mice which was not previously detected (61). This suggests that interactions between different microbial factors have complex and far-reaching effects on immune responses and disease onset and development. Host responses to periodontal infection are reflected in circulating antibodies against specific bacterial epitopes. In RA, such responses may sustain immune activation and favor ACPA production, while also offering potential value for patient stratification. From a therapeutic standpoint, controlling periodontal inflammation or limiting microbial triggers may complement RA management, especially in genetically susceptible individuals. When analyzing data of individual RA patients, whether they possess SE can also determine whether oral bacteria are pathogenic or promote ACPA generation only in patients with an HLA-DRB1 genetic background (62). These findings suggest that the interplay of genetics and environment allows microbial communities to trigger diverse autoimmune risks. Further investigation of susceptibility genes, including those related to immune regulation and metabolism, is essential to fully understand their impact on RA pathogenesis and to inform personalized treatment strategies.

### 4.2 Therapeutic factor

Pharmacological interventions used in RA management can directly influence the composition and balance of the oral microbiota, thereby affecting disease progression and treatment outcomes. The effects of drugs on microbiota are often individualized and may impact multiple microbial communities, including those in the oral cavity, gut, blood, and synovial fluid (63). Understanding how therapeutic agents alter microbiota composition is essential for optimizing personalized treatment strategies and reducing systemic inflammation. Dysbiosis within the gum or intestinal tissues can be reversed through appropriate treatment and may potentially recover within the blood and synovial tissues as well. In addition to adjusting microbial composition, many drugs used to treat RA can improve dysbiosis by reducing systemic inflammation and lowering intestinal permeability, which leads to a lower risk of additional microbiota spreading into synovial tissue. However, antibiotic use requires particular caution, as while it may reduce oral mucosal invasion, it can disrupt gut microbial balance and facilitate microbial translocation through the intestinal mucosa, potentially aggravating RA (64). This phenomenon has been illustrated in a case report by *Mukherjee et al.*, in which antibiotic-induced dysbiosis was associated with disease flare and systemic immune activation (27). Therefore, treatment strategies should carefully account for drug–microbiota interactions to achieve optimal clinical outcomes.

### 4.3 Smoking and gender in patients

**Figure 3:**
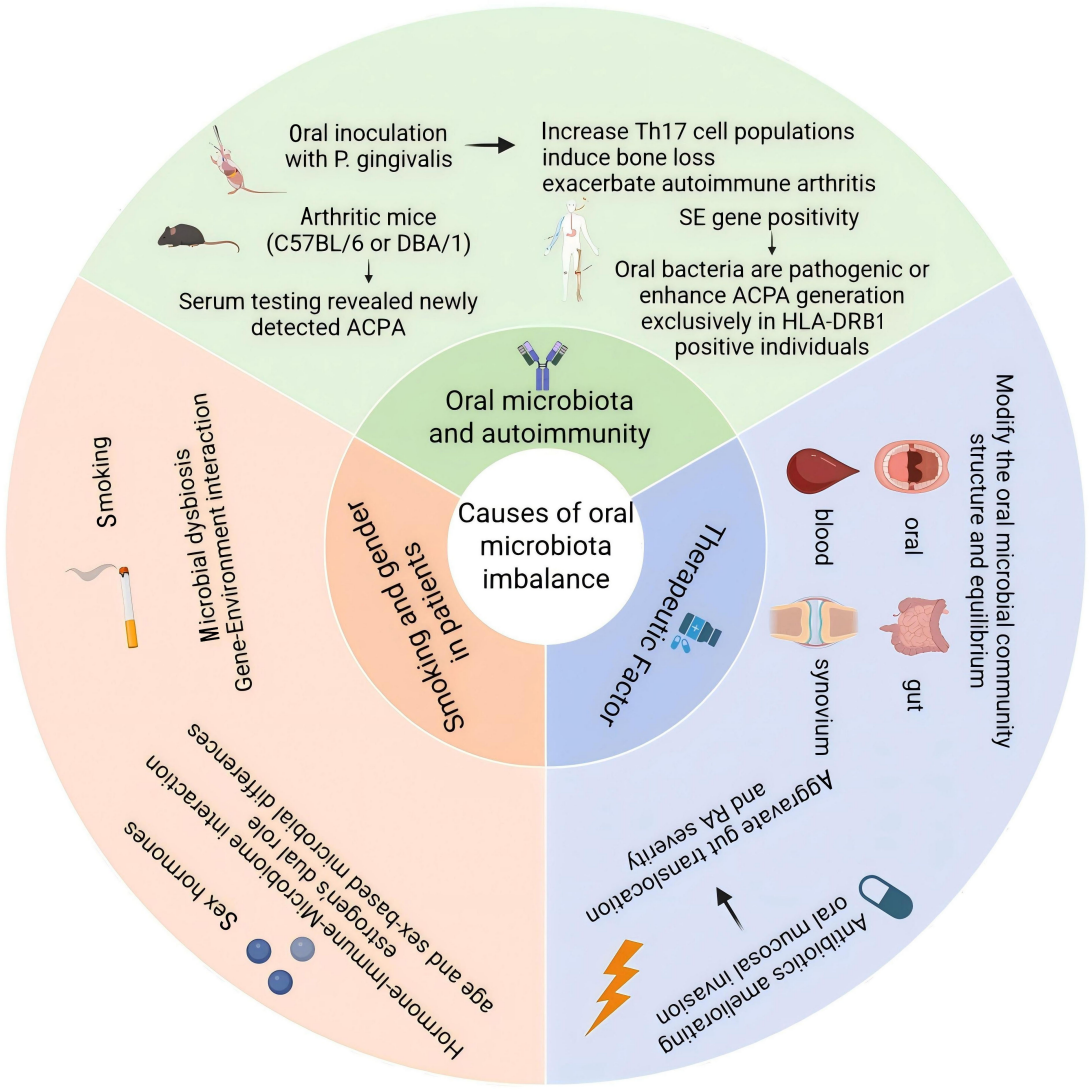
Key factors contributing to oral microbiota imbalance and RA development: (1) Autoimmune interaction: *P. gingivalis* promotes Th17 responses and ACPA production, especially in HLA-DR SE carriers; (2) Treatment effects: RA drugs modulate microbiota and inflammation, while antibiotics may worsen dysbiosis; (3) Environmental and biological factors: Smoking and sex hormones disrupt microbial balance and immune regulation, influencing RA risk. Created in https://BioRender.com.

Exposure to smoking has been shown to disrupt the balance of oral and gut microbiota and worsen the severity of RA. Smokers may carry a presumed periodontal pathogenic bacterial community. Early studies have shown that *P. gingivalis*, *A. actinomycetemcomitans*, and *Actinomyces* are more prevalent in smokers than non-smokers (65). Smoking may be a major environmental risk factor for the development of RA. There is strong evidence to suggest a robust gene-environment interaction between smoking and HLA-DR SE alleles. This gene-smoking interaction appears to have a significant impact on the development of ACPA^+^ RA (66). However, this interaction has not been confirmed in ACPA^-^ RA patients. It is currently unknown whether quitting smoking can affect the risk of developing RA in the future. There is an interaction between sex hormones and microbial habitats as well as host immune responses. Estrogen could regulate immune responses and participate in the onset of RA, which is why women are more dominant in RA. Furthermore, there are significant differences in the microbiota between men and women at different ages, which may also have a certain impact on the development of RA (67, 68) (**Figure 3**).

## 5 Pathway of oral microbiota leading to RA

### 5.1 Bloodstream transmission

When an individual suffers from periodontitis, various activities and circumstances can contribute to the spread of oral bacteria into the bloodstream, a condition known as bacteremia. The tearing of the epidermis within the periodontal pocket, which is often exacerbated by the inflammatory processes characteristic of periodontitis, provides a direct route for bacteria to enter the circulation (69). Routine oral hygiene practices, such as aggressive brushing and flossing, can further damage the delicate tissues of the gums, facilitating bacterial entry into the bloodstream. Invasive dental procedures, including tooth extraction, dental cleaning, and orthodontic treatment, also pose significant risks for bacteremia. These procedures often involve mechanical manipulation of the teeth and gums, leading to disruptions in the mucosal barrier and providing an entry point for oral pathogens.

Even routine activities like vigorous chewing or brushing can cause micro-abrasions in the oral mucosa, creating small but significant breaches that allow bacteria to escape from the oral cavity into the systemic circulation (70). Moreover, periodontitis itself contributes to this problem by promoting the vascularization of the periodontal pocket (71). The increased blood flow to the affected areas, combined with gum ulceration and the breakdown of tissue integrity, creates an ideal pathway for periodontal pathogens to enter the bloodstream. Once these bacteria gain access to the systemic circulation, they can disseminate throughout the body, potentially contributing to the development or exacerbation of systemic conditions such as RA, cardiovascular disease, and other inflammatory disorders (72). In addition to whole bacteria, periodontal pathogens can release extracellular vesicles that enter the bloodstream, carrying virulence factors, lipopolysaccharides, and immunogenic proteins capable of activating immune cells at distant sites (73). The connection between oral health and overall systemic health underscores the importance of managing periodontitis to preserve oral function while preventing its broader implications on general health.

### 5.2 Gastrointestinal tract

**Figure 4:**
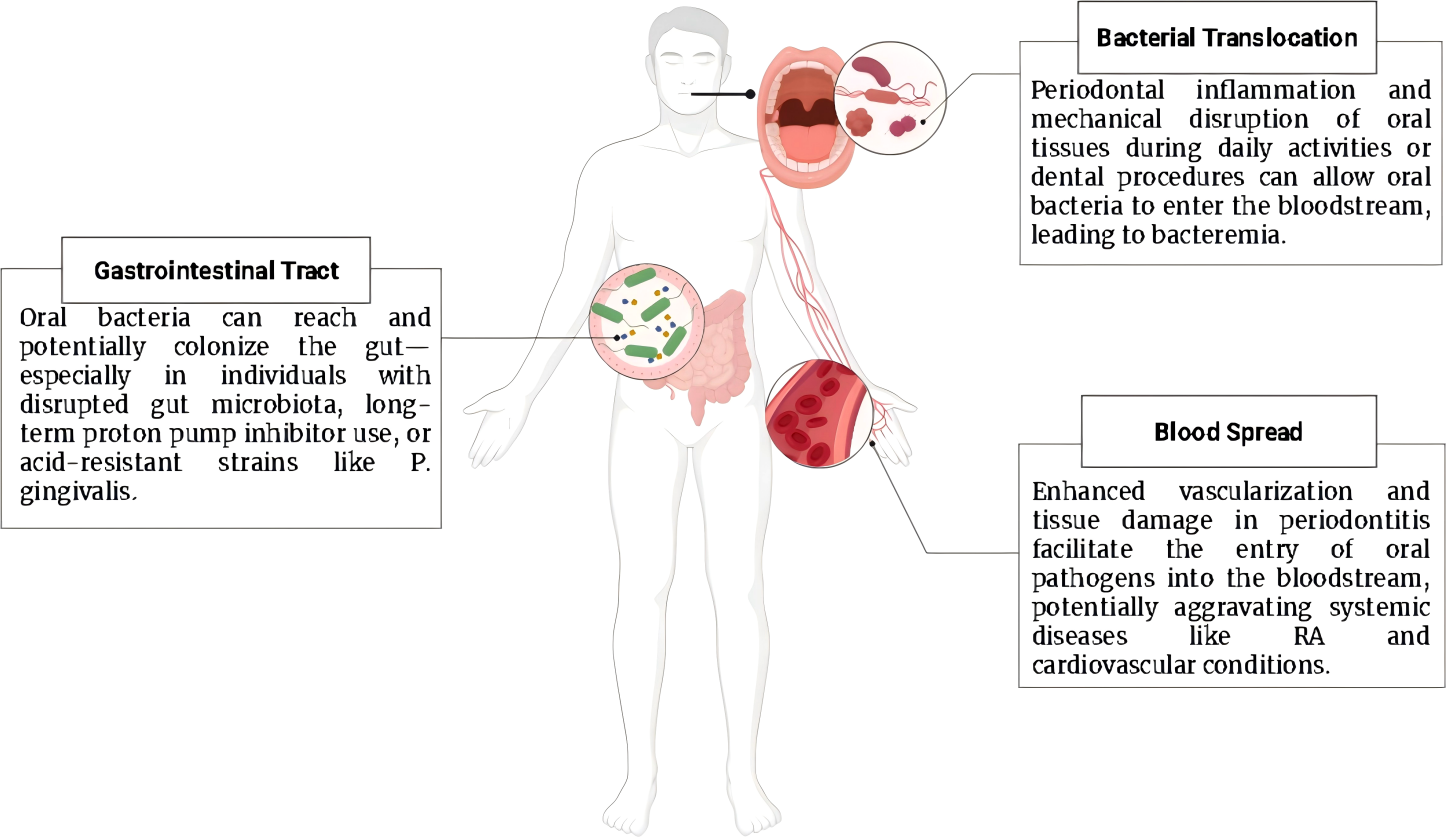
Pathway of oral microbiota leading to RA. Created in https://BioRender.com.

Humans ingest approximately 1.5 liters of saliva daily, which contains a diverse array of oral bacteria. As this saliva is swallowed, it serves as a pathway for oral microbiota to enter the gastrointestinal tract, potentially influencing gut health (74). Gastric acid and alkaline bile act as significant obstacles to the establishment of oral microbial communities in the intestine, sparking considerable discussion about the possibility of oral microbiota colonizing the gut via an internal pathway. A recent study showed that there was no evidence of oral bacteria colonizing the distal intestine in healthy adults; instead, additional evidence of oral to intestinal transmission was observed in patients with colorectal cancer and RA (75). Saliva contains mucoprotein, water, lipids, and proteins, which can protect microorganisms against being eliminated by stomach acid and allow them to survive in the gastrointestinal tract. It is estimated that patients with severe periodontitis swallow large amounts of *P. gingivalis* every day, which can alter the gut microbiota if it enters the intestines. Alterations in gut microbial composition can subsequently affect the production of short chain fatty acids (SCFAs), key microbial metabolites involved in maintaining intestinal barrier integrity and regulating immune responses (76). Reduced SCFA levels may promote intestinal permeability and systemic inflammation, thereby facilitating immune dysregulation relevant to RA. However, due to the barrier function of the gastrointestinal tract and its acidity, oral bacteria hardly reach and colonize a normal intestine. Nevertheless, these barriers may be compromised under the following three conditions through which oral bacteria can enter the intestines. Firstly, disrupting gut microbiota leads to increased colonization of oral bacteria. For example, antibiotic use could disrupt normal gut microbiota composition and facilitate colonization of oral bacteria in the intestines (77). Secondly, long-term use of proton pump inhibitors results in gastric dysfunction and a significant increase in colonization by oral bacteria such as *Actinomyces* species, *Streptococcus* species, and *Veillonella* species in the intestines. At the same time, gastritis or gastric surgery may also reduce exposure to ingested oral bacteria in gastric fluid (78). Thirdly, oral bacteria with strong acid resistance, such as *P. gingivalis*, can pass through the gastric barrier and enter the intestinal cavity (79). Analyzing the microbiota in the oral cavity, gut, blood, and synovium will help to better understand their relationship with RA. At the same time, in depth analysis of these contents may lead to improved methods for preventing and managing RA (**Figure 4**).

## 6 RA with periodontitis: therapeutic interventions

### 6.1 Non-surgical periodontal treatment

Non-surgical periodontal treatment (NSPT), Non-surgical periodontal treatment (including supra-gingival scaling, sub-gingival curettage, and intensified oral hygiene guidance, is a standard measure for periodontal management and is also the most commonly adopted intervention for patients with RA and periodontitis. NSPT can significantly improve periodontal outcomes such as probing depth, attachment loss, and the gingival bleeding index (80). More importantly, in the short term follow-up period for RA patients who received neuroendocrine therapy, their systemic inflammatory indicators (such as disease activity scores and acute-phase reactants) showed a downward trend. However, these systematic benefits are usually only effective within a follow-up period of 3 to 6 months, and their sustainability has not yet been confirmed through long term studies (81). These observations highlight that adjunctive therapeutic strategies may influence long-term systemic outcomes in RA patients with periodontitis. Therefore, the existing evidence supports NSPT as a basic and safe intervention method, but its effect in improving the systemic activity of RA still needs to be verified by larger scale, long-term follow-up randomized studies.

### 6.2 Antibacterial adjunctive therapy

In periodontal clinical practice, the short course systemic application of amoxicillin combined with metronidazole is a common strengthening measure for non-surgical treatment, especially for patients with aggressive or refractory periodontitis. Relevant studies have shown that this combined medication can further reduce the population of red stained complex bacteria and improve the periodontal clinical outcomes after non-surgical treatment (82). In small sample trials and case series of RA patients with severe periodontitis, some patients showed a trend of accompanying reduction in RA disease activity after receiving such intensified treatment, in addition to the improvement of periodontal parameters (83). In contrast, a case report by *Mukherjee et al.* described RA exacerbation following systemic antibiotic exposure, which was associated with marked gut microbial dysbiosis and increased systemic immune activation (27). This case underscores that antibiotic induced alterations of the gut microbiota may offset periodontal benefits and adversely affect RA disease activity in susceptible individuals. However, this conclusion lacks the support of large-sample randomized controlled trials, and the existing data are still insufficient to consider it as a routine recommendation. Moreover, systemic antibiotic treatment needs to consider the risk of drug resistance and individual tolerance. Therefore, antibacterial adjunctive therapy should be considered an individualized option for severe or refractory periodontitis in RA patients, with careful evaluation of potential systemic effects related to microbiota disruption.

### 6.3 The influence of anti-rheumatic drugs on periodontal status

Apart from the potential improvement of RA through periodontal intervention, the rheumatic disease drugs themselves may also indirectly improve the periodontal condition by controlling systemic inflammation. Studies have shown that biological agents such as anti-TNF and anti-IL-6 are associated with a decrease in periodontal inflammation indicators in some patients, suggesting that the alleviation of systemic inflammation may bring benefits to periodontal health (84). However, the existing evidence is not entirely consistent. Studies have pointed out that certain combination regimens (such as TNF inhibitors combined with methotrexate) are associated with an increase in gingivitis levels (85). This suggests that different drug categories and combination therapy methods may have differential effects on the oral inflammatory microenvironment. Overall, the existing data support the view that RA control and periodontal health may have a bidirectional benefit, but the mechanism is complex and heterogeneous, and clinical studies with periodontal parameters as the primary endpoint need to be designed to clarify the real impact of anti-rheumatic drugs on oral health.

### 6.4 Lifestyle intervention and long-term maintenance

In addition to specific periodontal treatments and RA medications, lifestyle interventions and long-term maintenance are also equally important. There is a consensus that good oral hygiene behaviors (such as regular brushing, the use of dental floss, and interdental brushes), quitting smoking, and regular professional maintenance can significantly reduce the risk of PD recurrence. For patients with RA, incorporating oral health education and long-term follow-up can help reduce local inflammatory burden, thereby reducing systemic inflammatory levels to a certain extent (86). Multidisciplinary collaboration is the key to improving therapeutic efficacy: the joint management of rheumatology physicians and periodontology physicians can achieve information sharing on the disease and coordinated treatment plans, avoid treatment blind spots, and promote dual control of RA and PD. Future clinical studies should also set periodontal and rheumatoid outcomes as a common endpoint, so as to more comprehensively evaluate the clinical significance of the intervention.

## 7 Discussion

In recent years, the interaction between the oral microbiota and RA has become an increasingly prominent research focus. Evidence from epidemiology, clinical, and mechanistic studies indicates that the dysregulation of the oral microbiota accompanies the onset of RA and may further act as a potential trigger and amplifier of systemic autoimmune responses (87, 88). This review focuses on several key aspects of this interaction, including microbial induced citrullination, host genetic susceptibility, and the immunological dysregulation phenomenon linking periodontitis and RA. One of the core findings is that citrullination and the generation of ACPAs serve as a key mechanism linking oral pathogens to RA. Among the identified microbial factors, PPAD produced by *P. gingivalis* represents a unique pathogenic feature, as it directly catalyzes the citrullination of both bacterial and host proteins in a calcium independent manner. This enzymatic activity provides a sustained source of neo-epitopes that can be presented by HLA-DRB1 shared epitope molecules, thereby facilitating ACPA generation and loss of immune tolerance. In parallel, LtxA from *A. actinomycetemcomitans* induces hypercitrullination in neutrophils by triggering calcium influx and PPAD activation, suggesting a convergent pathogenic pathway mediated by distinct oral bacteria. *P. gingivalis* is the most significant oral pathogen because its unique PPAD enzyme citrullinates both bacterial and host proteins (89). This process helps to break immune tolerance and promotes the generation of ACPAs, especially in genetically susceptible individuals carrying HLA-DRB1 common allele loci. Similarly, *A. actinomycetemcomitans* induces hypercitrullination in host neutrophils through the action of LtxA, indicating that multiple microbial species may follow the same pathogenic pathway (90). These findings emphasize a mechanistic link between oral microbiota imbalance and systemic autoimmunity. However, it should be noted that not all clinical and microbiome studies have consistently identified enrichment of *P. gingivalis* or *A. actinomycetemcomitans* in RA cohorts. Some reports have failed to demonstrate a direct association between their abundance and disease activity or autoantibody levels, possibly due to differences in population background, sampling sites, disease stage, or sequencing approaches. Another key point is the bidirectional relationship between periodontitis and RA. Patients with RA have a higher prevalence and severity of periodontitis, and chronic periodontal inflammation can exacerbate the condition of RA through systemic dissemination of bacterial products and pro-inflammatory mediators (91). Common immunopathological mechanisms, particularly those involving Th17 responses, B cell activation, and RANKL mediated osteoclastogenesis, emphasize the role of a common inflammatory axis in causing destruction of joints and periodontal tissues (92). This interaction may explain why treatment control of one disease sometimes leads to improvement of the other.

The pathways related to how oral bacteria affect systemic diseases remain an area of ongoing research. The blood dissemination of oral pathogens, especially during bacteremia caused by routine oral activities or dental procedures, provides a possible mechanism (93). Additionally, the colonization of oral microbiota through swallowing and its possible establishment in the gastrointestinal tract may represent another pathway, especially in cases of impaired gastric barrier function or dysbiosis of the intestinal microbiota (94). These mechanisms emphasize that RA should not be viewed solely as an autoimmune disease but rather as a disease influenced by the microbiota ecology in multiple systemic sites. Within this framework, PAD and LtxA mediated hypercitrullination may represent critical molecular links connecting mucosal inflammation to systemic autoimmunity, particularly in genetically susceptible hosts. These pathways provide a mechanistic explanation for how localized periodontal dysbiosis can initiate or amplify joint specific immune responses. From a therapeutic perspective, current evidence suggests that periodontal treatment may have systemic benefits for patients with RA. Non-surgical periodontal treatment has consistently improved local periodontal indicators and is associated with a reduction in systemic inflammatory markers, although its impact on the disease activity of RA is inconsistent and often temporary (95). These inconsistent findings may be explained by heterogeneity in study design, limited sample sizes, variation in follow up duration, baseline RA disease activity, and differences in concomitant use of disease modifying anti-rheumatic drugs. Adjuvant antibiotic treatment shows promise in certain severe cases, but its long-term benefits and risks must be carefully weighed. Anti-rheumatic drugs may also indirectly affect periodontal health by reducing systemic inflammation, although the specific impact of the drugs on the oral microenvironment is diverse (96). It is important to note that lifestyle interventions such as smoking cessation and strict oral hygiene should not be overlooked, as they may simultaneously reduce both periodontal and systemic inflammation burdens.

Despite these insights, there are still some limitations. It is not clear whether specific oral pathogens are direct initiators of RA or primarily act as amplifiers of existing immune dysregulation. Although genetic susceptibility seems to influence the pathogenicity of microorganisms, especially in HLA-DRB1 SE carriers, the precise host-microbiome interactions leading to clinical RA still need to be further clarified. Most studies on the clinical effects of periodontal treatment in RA are limited by small sample sizes, short follow-up periods, and inconsistent treatment protocols, making it difficult to draw clear conclusions about the long-term systemic benefits. Notably, microbial drivers such as PPAD from *P. gingivalis* and LtxA from *A. actinomycetemcomitans* act as key pathogenic factors by promoting excessive protein citrullination and subsequent autoimmune activation. This highlights that defined bacterial virulence mechanisms, rather than general dysbiosis alone, are critical in shaping RA-associated immune responses. Overall, the intricate interactions among oral microbiota, host immune mechanisms, and genetic susceptibility are deeply involved in the pathogenesis of RA. *P. gingivalis* and *A. actinomycetemcomitans* contribute to systemic autoimmunity by inducing protein citrullination, activating pro-inflammatory cytokines, and facilitating microbial translocation. This triad of mechanisms, mucosal inflammation, immune activation, and systemic dissemination creates a pathogenic link between the oral cavity and joint tissues. Recognizing this connection underscores the importance of integrated healthcare approaches that consider oral health as a fundamental aspect of systemic disease management. Future research should continue to delve deeper into these mechanisms and explore therapeutic approaches targeting the microbiome, applying them as part of a comprehensive treatment strategy for RA.

## 8 Summary and future perspectives

The available evidence strongly supports a biological intersection between oral microbiota and RA. Oral pathogens, particularly *P. gingivalis* and *A. actinomycetemcomitans*, may initiate or intensify systemic autoimmunity through citrullination and immune dysregulation, while periodontitis acts as a chronic inflammatory burden that aggravates RA progression. Although causal relationships remain incompletely defined, addressing oral health as part of RA management holds significant promise for improving patient outcomes. Future research should focus on longitudinal and mechanistic studies integrating microbiome profiling, immunological assays, and genetic analyses to better define causal pathways. In addition, well designed randomized controlled trials are necessary to evaluate the impact of comprehensive periodontal management on RA outcomes. Therapeutic approaches targeting the microbiome, such as PPAD inhibitors, probiotics or microbiota regulators, may open up new avenues for disease treatment. Ultimately, a multidisciplinary management strategy that incorporates both rheumatologic and periodontal care may offer the greatest benefit to patients.

## Glossary

RA: Rheumatoid arthritis
ACPA: Anti-citrullinated protein antibody
DC: Dendritic cell
APC: Antigen-presenting cell
IL: Interleukin
NET: Neutrophil extracellular trap
LPS: Lipopolysaccharide
SE: Shared epitope
*P. gingivalis*: *Porphyromonas gingivalis*
ACPA^-^: ACPA negative
RANKL: Receptor activator of nuclear factor kappa-B ligand
LtxA: Leukotoxin A
*P. intermedia*: *Prevotella intermedia*
BspA: Bacteroides surface protein A
NSPT: Non-surgical periodontal treatment
PD: Periodontal disease
RF: Rheumatoid factor
NK: Natural killer
TNF-α: Tumor necrosis factor-alpha
ROS: Reactive oxygen species
TLR: Toll-like receptor
HLA: Human leukocyte antigen
ACPA^+^: ACPA positive
PPAD: Peptidylarginine deiminase
MMP: Metalloproteinases
*A. actinomycetemcomitans*: *Aggregatibacter actinomycetemcomitans*
*F. nucleatum*: *Fusobacterium nucleatum*
*T. forsythia*: *Tannerella forsythia*
NLRP3: NOD-like receptor family pyrin domain containing 3
SCFAs: Short-chain fatty acids
